# Supplementary material for: Changes in Phenylacetylglutamine Levels Provide Add-On Value in Risk Stratification of Hypertensive Patients: A Longitudinal Cohort Study
Source: Metabolites. 2025 Jan 20;15(1):64. doi: 10.3390/metabo15010064 (PMC11767660; doi:10.3390/metabo15010064)

Supplementary Materials

Changes phenylacetylglutamine level provide add-on value on risk stratification in hypertensive patients: a longitudinal cohort study

Table S1: The detailed parameters of targeted MS instrument and LC gradient 2

Table S2: The detailed VIF and 1/VIF of all covariates in the discovery cohort and validation cohort...3

Table S3: Multivariate-adjusted hazard ratios for primary endpoint associated with plasma concentrations of PAGln, excluding the highest and lowest 2.5% of PAGln data.......................................4

Table S4: Multivariate-adjusted hazard ratios for primary endpoint associated with plasma concentrations of PAGln, with cardiac events...4

Table S5. Multivariate-adjusted hazard ratios for primary endpoint associated with plasma concentrations of PAGln, with cerebrovascular events and cardiac events..5

Table S6: Multivariate-adjusted hazard ratios for primary endpoint associated with plasma concentrations of PAGln, with available data...5

Figure S1: Detailed inclusion and exclusion criteria..6

Figure S2: The ROC curve in discovery cohort...7

Supplemental Table 1. The detailed parameters of targeted MS instrument and LC gradient

**Xevo TQ-S micro**	**PAGln**	**PAGln-d5**
Mode	Positive	
Desolvation temperature (°C)	350	
Desolvation gas flow (L/Hr)	650	
Capillary voltage (kV)	16	14
Cone voltage (V)	40	40
**LC Condition**		
Column	ACQUITY UPLC BEH HILIC (2.1 mm inner diameter×100mm with 1.7-μm particles, Warters)	
Column Chamber T (°C)	40°C	
Flow Rate	0.4mL/min	
Mobile Phase A	10mM ammonium formate-0.1% formic acid aqueous solution	
Mobile Phase B	0.1% formic acid acetonitrile mixed solution	
Gradient (B %)	0.0 min-95%<br>4.0 min-50%<br>4.1 min-95%<br>5.0 min-stop	

Supplemental Table 2. The detailed VIF and 1/VIF of all covariates in the discovery cohort and validation cohort

Control variables	Discovery cohort		Validation cohort	
	VIF	1/VIF	VIF	1/VIF
**sex**	1.73	0.578043	1.61	0.622799
**age**	1.46	0.683054	1.26	0.796461
**smoking status**	1.57	0.638651	1.54	0.647623
**CHD**	1.15	0.869524	1.16	0.86286
**diabetes status**	1.18	0.846140	1.12	0.895758
**TC**	2.77	0.360761	2.50	0.400377
**LDL**	2.91	0.360761	2.46	0.406127
**ARB**	1.21	0.828020	1.05	0.953858
**CCB**	1.02	0.976250	1.04	0.962902
**ACEI**	1.08	0.923404	1.04	0.963077
**β-Blocker**	1.15	0.976250	1.03	0.972297

Abbreviations: CHD, coronary heart disease; TC, total cholesterol; LDL, low-density lipoprotein; ACEI, an-giotensin converting enzyme inhibitor; ARB, angiotensin receptor blocker; CCB, calcium channel blocker

Supplemental Table 3. Multivariate-adjusted hazard ratios for primary endpoint associated with plasma concentrations of PAGln, excluding the highest and lowest 2.5% of PAGln data

Plasma PAGln(μmol/L)	crude model		Multivariable 1[1]		Multivariable 2[2]		Multivariable 3[3]	
	HR[95% CI]	p Value	HR[95% CI]	p Value	HR[95% CI]	p Value	HR[95% CI]	p Value
PAGln<1.047	1.0[referent]		1.0[referent]		1.0[referent]			
PAGln≥1.047	2.69[1.86-3.89]	< 0.001	1.73[1.17-2.56]	0.006	1.83[1.23-2.73]	0.003	1.87[1.25-2.78]	0.002

Hazard ratios (HRs) and 95% CI were estimated by plasma PAGln levels.

[1]Multivariable 1 was adjusted for sex and age.

[2]Multivariable 2 was adjusted for sex, age, CHD, smoking status, diabetes, LDL, TC.

[3]Multivariable 3 was adjusted for sex, age, CHD, smoking status, diabetes, LDL, TC, ACEI, β-Blocker, CCB, ARB

Supplemental Table 4. Multivariate-adjusted hazard ratios for primary endpoint associated with plasma concentrations of PAGln, with cardiac events

Plasma PAGln(μmol/L)	crude model		Multivariable 1[1]		Multivariable 2[2]		Multivariable 3[3]	
	HR[95% CI]	p Value	HR[95% CI]	p Value	HR[95% CI]	p Value	HR[95% CI]	p Value
PAGln<1.047	1.0[referent]		1.0[referent]		1.0[referent]			
PAGln≥1.047	3.02[1.99-4.59]	< 0.001	2.07[1.33-3.23]	0.001	2.16[1.38-3.38]	0.001	2.21[1.41-3.46]	0.001

Hazard ratios (HRs) and 95% CI were estimated by plasma PAGln levels.

[1]Multivariable 1 was adjusted for sex and age.

[2]Multivariable 2 was adjusted for sex, age, CHD, smoking status, diabetes, LDL, TC.

[3]Multivariable 3 was adjusted for sex, age, CHD, smoking status, diabetes, LDL, TC, ACEI, β-Blocker, CCB, ARB

Supplemental Table 5. Multivariate-adjusted hazard ratios for primary endpoint associated with plasma concentrations of PAGln, with cerebrovascular events and cardiac events

Plasma PAGln(μmol/L)	crude model		Multivariable 1[1]		Multivariable 2[2]		Multivariable 3[3]	
	HR[95% CI]	p Value	HR[95% CI]	p Value	HR[95% CI]	p Value	HR[95% CI]	p Value
PAGln<1.047	1.0[referent]		1.0[referent]		1.0[referent]			
PAGln≥1.047	2.42[1.64-3.57]	< 0.001	1.67[1.11-2.52]	0.015	1.75[1.16-2.66]	0.008	1.80[1.19-2.74]	0.006

Hazard ratios (HRs) and 95% CI were estimated by plasma PAGln levels.

[1]Multivariable 1 was adjusted for sex and age.

[2]Multivariable 2 was adjusted for sex, age, CHD, smoking status, diabetes, LDL, TC.

[3]Multivariable 3 was adjusted for sex, age, CHD, smoking status, diabetes, LDL, TC, ACEI, β-Blocker, CCB, ARB

Supplemental Table 6. Multivariate-adjusted hazard ratios for primary endpoint associated with plasma concentrations of PAGln, with available data

Plasma PAGln(μmol/L)	crude model		Multivariable 1[1]		Multivariable 2[2]		Multivariable 3[3]	
	HR[95% CI]	p Value	HR[95% CI]	p Value	HR[95% CI]	p Value	HR[95% CI]	p Value
PAGln<1.047	1.0[referent]		1.0[referent]		1.0[referent]			
PAGln≥1.047	3.17[2.15-4.66]	< 0.001	1.94[1.28-2.92]	0.002	2.00[1.31-3.04]	0.001	2.08[1.37-3.16]	0.001

Hazard ratios (HRs) and 95% CI were estimated by plasma PAGln levels.

[1]Multivariable 1 was adjusted for sex and age.

[2]Multivariable 2 was adjusted for sex, age, CHD, smoking status, diabetes, LDL, TC.

[3]Multivariable 3 was adjusted for sex, age, CHD, smoking status, diabetes, LDL, TC, ACEI, β-Blocker, CCB, ARB

Supplemental Figure 1. Detailed inclusion and exclusion criteria

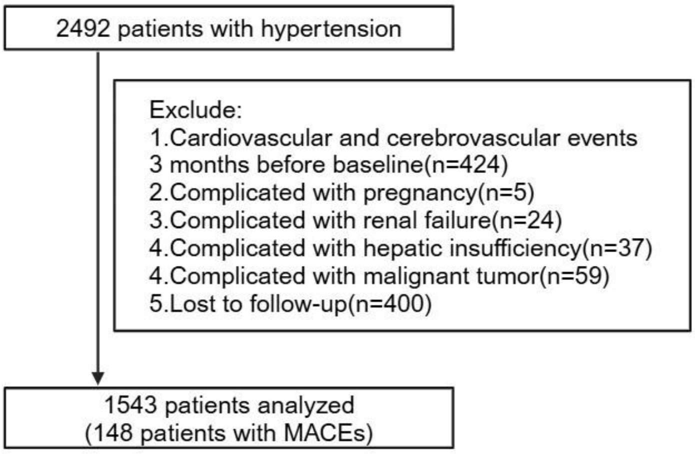

Flow chart of study inclusion/exclusion criteria

Supplemental Figure 2. The ROC curve in discovery cohort

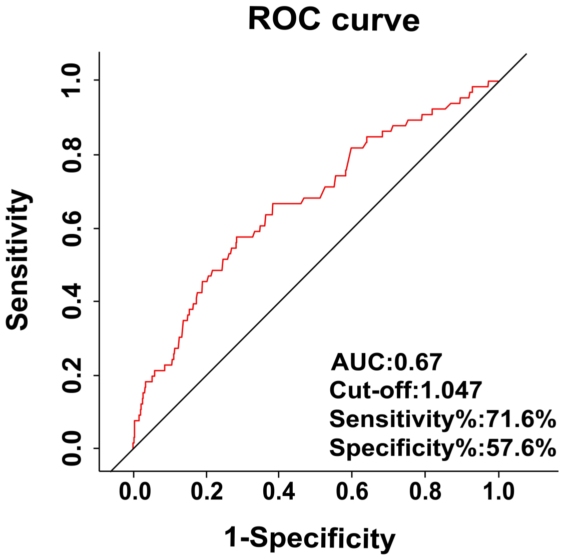

Supplement: Supplementary file 1 [file metabolites-15-00064-s001.zip › metabolites-3393238-supplementary.pdf]
